# Guided piezoelectric decompression of the inferior alveolar canal following hydraulic calcium silicate-based sealer extrusion: a case report

**DOI:** 10.1186/s12903-026-08333-3

**Published:** 2026-04-24

**Authors:** Hesham Elhawary, Reham Hassan

**Affiliations:** 1 Faculty of Dentistry, Cairo University, Cairo, Egypt; 2 Faculty of Oral and Dental Medicine, Future University in Egypt, Cairo, Egypt

**Keywords:** Inferior alveolar nerve injury, Bioceramic sealer extrusion, Digitally guided surgery, Piezoelectric osteotomy, Nerve decompression, CBCT, L-PRF.

## Abstract

**Background:**

To describe the diagnosis and surgical management of an inferior alveolar nerve (IAN) injury caused by extrusion of a bio-ceramic endodontic sealer into the mandibular canal using digitally guided piezoelectric decompression.

**Case presentation:**

A 12-year-old female presented with persistent right-sided anesthesia of the lip and the chin following endodontic treatment of tooth #46 (total loss of sensation). Clinical neurosensory examination and cone-beam computed tomography (CBCT) confirmed extrusion of sealer material into the inferior alveolar canal. Virtual surgical planning was performed, and a CAD/CAM patient-specific surgical guide was fabricated. Guided piezoelectric corticotomy was used to access the canal and remove the extruded material. Leukocyte- and platelet-rich fibrin (L-PRF) was applied for nerve coverage, followed by reconstruction with autologous fibrin-stabilized bone graft (sticky bone).

Postoperative follow-up demonstrated gradual improvement in neurosensory function. Electric pulp testing (EPT) values of adjacent teeth changed from elevated thresholds (55–76) to values within normal limits (12–34) over a 3-month period. Follow-up CBCT confirmed complete removal of the extruded material and re-establishment of the inferior alveolar canal space.

**Conclusions:**

Digitally guided piezoelectric decompression enabled controlled access to the inferior alveolar canal and removal of extruded bio-ceramic sealer in a case of persistent IAN dysfunction.

**Supplementary Information:**

The online version contains supplementary material available at 10.1186/s12903-026-08333-3.

## Background

Injury to the inferior alveolar nerve is a recognized complication of oral surgical procedures, particularly mandibular third molar removal and implant placement; however, endodontic material extrusion represents an additional, less frequent aetiology [1]. Bio-ceramic sealers (BCS) are generally considered bio-compatible within the confines of the root canal system, yet extrusion beyond the apical foramen may result in adverse neural effects related to alkalinity, setting expansion, or direct mechanical compression within the mandibular canal [2, 3].

When neurosensory disturbance persists and radio-graphic imaging confirms involvement of the inferior alveolar canal, surgical intervention may be indicated to relieve compression and reduce the risk of permanent nerve dysfunction [4]. The use of digital surgical planning, CAD/CAM-generated guides, and piezoelectric bone surgery allows precise localization and selective osteotomy in proximity to neural structures [5, 6].

## Case presentation

This case report was prepared in accordance with the Preferred Reporting Items for Endodontic Case Reports (PRICE) 2020 guidelines [7]. A 12-year-old female was referred one month after completion of root canal treatment of tooth #46, reporting persistent right-sided anesthesia (numbness) of the lip and the chin. Former root canal treatment involved mechanical instrumentation to an apical size of #35/0.04 and obturation using a premixed calcium-silicate-based bioceramic sealer (Ceraseal; Meta Biomed, Cheongju, South Korea) and a single-cone technique. Previous conservative management included systemic corticosteroids and vitamin B12 supplementation, without reported improvement.

### Clinical examination

Neurosensory testing using light touch sensation (cotton wisp), two-point discrimination (standardized dental probe), and thermal testing demonstrated reduced tactile perception within the distribution of the right inferior alveolar nerve. The patient’s neurosensory deficit was evaluated using a 0–10 scale, where 0 represented total anesthesia and 10 represented normal sensation. The patient exhibited a significantly reduced response (Visual Analog Scale score of 0/10).Electric pulp testing of adjacent teeth revealed elevated response thresholds ranging from 55 to 76, suggestive of altered neural conduction [8].

### Radiographic findings

**Fig. 1:**
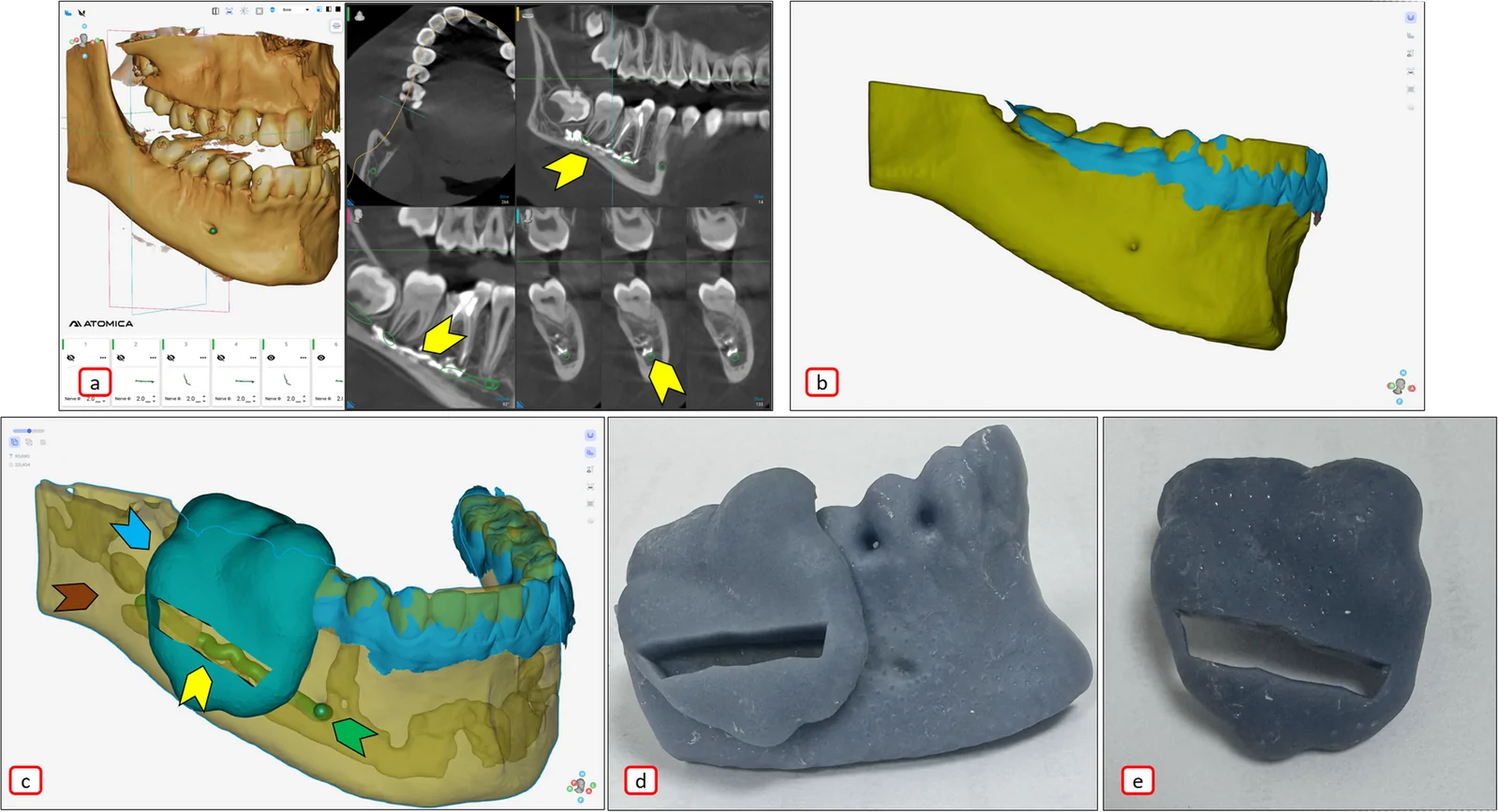
Diagnostic Imaging and Preoperative Virtual Planning. **a **Preoperative cone-beam computed tomography (CBCT) panoramic reconstruction showing radiopaque bioceramic sealer (arrow) extruded into and occluding the course of the right inferior alveolar canal. **b **Superimposition of the intraoral digital scan onto the CBCT data-set for integrated three-dimensional treatment planning. **c** Virtual design of the patient-specific, tooth- and bone-supported surgical guide in the planning software (highlighted with blue arrow), outlining the planned osteotomy window (highlighted with yellow arrow). **d** Three-dimensional bone model demonstrating the planned relationship of the surgical guide to the lateral cortex of the mandible and the underlying inferior alveolar canal. **e** The finalized surgical guide fabricated via stereolithography (SLA) 3D printing in a medical-grade resin

CBCT imaging demonstrated radiopaque material consistent with endodontic sealer within the inferior alveolar canal, in direct continuity with the apex of tooth #46 **(**Fig. 1a**)**.

### Diagnosis

Inferior alveolar nerve Axonotmesis (Sunderland Grade II/III) [9] secondary to extrusion of bio-ceramic endodontic sealer.

### Treatment planning and surgical procedure

CBCT data were imported into digital planning software (Atomica; Atomica Technology, Inc., Atlanta, GA, United States) to design a tooth- and bone-supported surgical guide defining the position and extent of the osteotomy window (Fig. 1b-e). The guide was designed by superimposing CBCT DICOM files and intraoral STL scans. The finalized design was fabricated via stereolithography (SLA) 3D printing using medical-grade resin”.

**Fig. 2:**
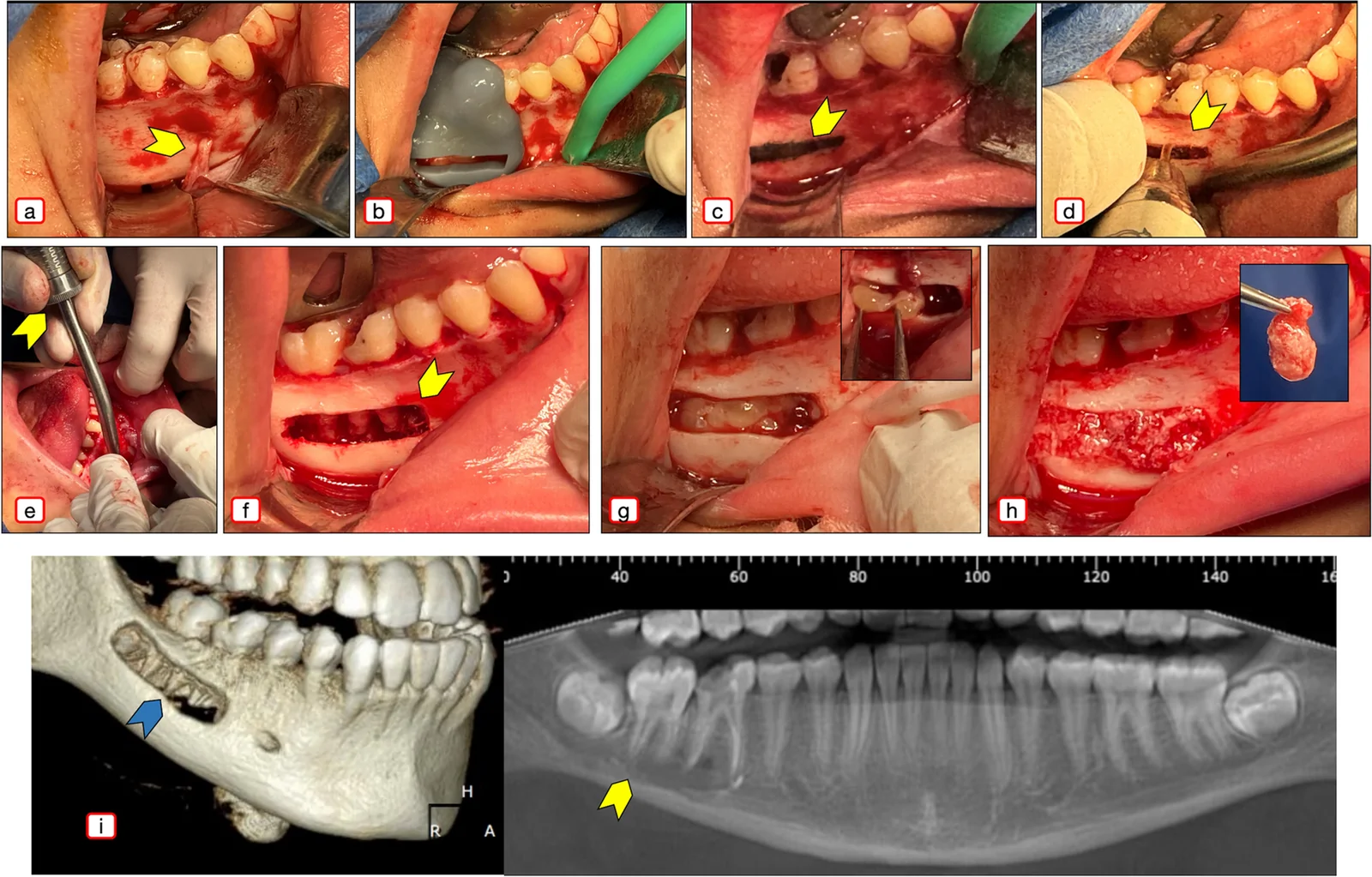
Intraoperative Procedural Steps and follow up CBCT: **a** Initial surgical exposure via a pyramidal mucoperiosteal flap. The mental nerve (arrow) has been identified. **b **Seating of the sterilized patient-specific surgical guide onto the dentition and lateral mandibular cortex. **c** Definitive marking of the osteotomy margins on the bone through the apertures of the surgical guide using a sterile surgical pen. **d** Execution of the buccal corticotomy using a piezoelectric surgical insert, ensuring selective bone cutting. **e** Collection of autogenous bone particulates (autograft) via a bone trap suction device during piezoelectric osteotomy. **f** The surgical field after complete nerve debridement. **g** Placement of leukocyte- and platelet-rich fibrin (L-PRF) membranes around the decompressed inferior alveolar nerve. **h** Final graft placement: the buccal defect has been filled with a "sticky bone" graft prior to flap closure. **i** Follow-up cone-beam computed tomography (CBCT) panoramic reconstruction obtained one month postoperatively. The image confirms the absence of the previously noted radiopaque sealer material within the right inferior alveolar canal (arrow), indicating successful surgical debridement. The bony outline of the canal appears patent

Surgery was performed one month after the initial endodontic treatment, following the failure of conservative management. Under general anaesthesia, guided piezoelectric corticotomy (Woodpecker; Guilin Woodpecker Medical Instrument Co., Ltd., China). was performed to access the mandibular canal. The cortical bone segment was removed, and the bone was collected by surgical bone collector suction tip (Medflair; Medflair Instruments Company, Sialkot, Pakistan), and the extruded sealer was carefully detached and removed from the vicinity of the nerve using Woodpecker Piezo Surgical Tip no. uc1 (Woodpecker; Guilin Woodpecker Medical Instrument Co., Ltd., China), Α distinct resistance was felt when the tip encountered the set Ceraseal material, which required a light ‘brushing’ motion to avoid nerve trauma (Fig. 2a-f).

L-PRF membranes were placed circumferentially around the exposed inferior alveolar nerve to provide soft tissue coverage (Fig. 2g) [10, 11]. Reconstruction of the buccal cortical defect was performed using autologous fibrin-stabilized bone graft (sticky bone) consisting of collected autogenous bone particles combined with allogeneic bone and platelet-derived concentrates (Fig. 2h) [12, 13].

Improvement in neurosensory symptoms was reported during the early postoperative period. Serial EPT measurements demonstrated a progressive reduction in response thresholds, reaching values within the normal range (12–34) by 3 months postoperatively (Table 1). Follow-up CBCT confirmed complete removal of the extruded material, patency of the inferior alveolar canal, and stability of the reconstructed bone (Fig. 2i).

**Table 1:** Serial Electric Pulp Test (EPT) readings

Tooth (FDI)	Preoperative	1 Week	1 Month	2 Months	3 Months
#41	62	58	45	28	18
#42	58	55	42	25	15
#43	55	52	38	22	12
#44	68	65	50	35	25
#45	76	72	60	40	32
#47	70	68	55	38	34

*Scale: 1–39 (vital), 40–79 (partial non-vitality), 80 (non-vital).*Numerical values represent the threshold of stimulation; decreasing values indicate returning sensitivity

## Discussion and conclusions

**Fig. 3:**
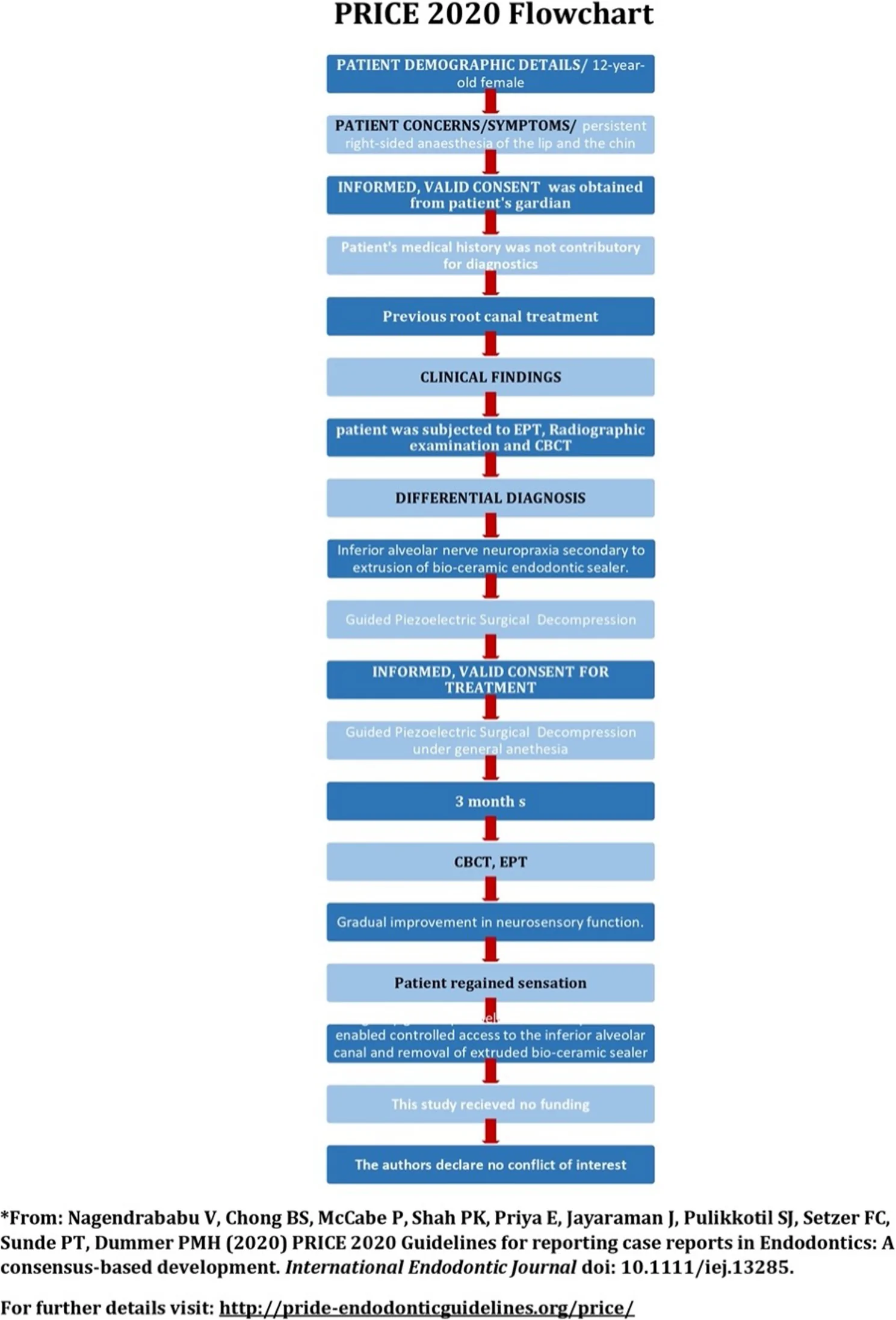
PRICE 2020 flowchart

A 12-year-old female presented with persistent right-sided anesthesia of the lip and the chin following endodontic treatment of tooth #46 secondary to extrusion of bio-ceramic endodontic sealer (Fig. 3). Clinical experience with these materials reveals a fundamental conflict; while their calcium-silicate composition is highly osteogenic and bio-compatible within peri-apical bone, they prove fundamentally non-neuro-compatible when in direct contact with neural tissue. This discrepancy arises because the extreme alkalinity (pH 12.0–12.8) required for bio-mineralization, which facilitates bone healing but triggers immediate chemical saponification of neural lipids induces a severe chemical burn on the epineurium, leading to protein denaturation and Wallerian degeneration(14–16). Beyond this chemical insult, the engineered high flow-ability and small particle size of premixed sealers facilitate their unintended transition into the low-resistance space of the inferior alveolar canal. Once situated within these rigid bony confines, any setting expansion exerts mechanical pressure and localized ischemia on the nerve. Unlike traditional materials, the ceramic-like set of BCS bonds to the environment, making surgical decompression exceptionally difficult and often resulting in permanent sensory deficits [14, 15].

The neurotoxic profile of bio-ceramic sealer is further complicated by formulation additives and the disruption of cellular homeostasis. The rapid release of calcium ions interferes with the delicate signaling required for nerve conduction, potentially causing the immediate of action potentials seen in clinical complete anesthesia cases.

Although some premixed sealers utilize vehicles such as Dimethyl Sulfoxide (DMSO) to enhance flowability—which may further increase membrane permeability to toxic ions [16] —CeraSeal is a DMSO-free formulation. Thus, the neural insult in this specific case is primarily attributed to the high caustic pH and the mechanical compression exerted by the material within the rigid confines of the mandibular canal. Unlike traditional endodontic sealers, the ceramic-like set of BCS bonds tightly to its surroundings, making surgical removal exceptionally challenging and often resulting in permanent sensory deficits if not addressed promptly. From an endodontic perspective, prevention through accurate working length determination, controlled obturation techniques, and careful case selection remains the primary strategy for avoiding such complications.

Management of inferior alveolar nerve disturbance following endodontic treatment is typically conservative in the initial phase. Pharmacologic therapy, observation, and serial neurosensory assessment are widely recommended, particularly in cases where imaging does not demonstrate direct canal occupation or progressive neurological deficit [1, 4]. Surgical intervention is therefore generally reserved for cases showing persistent or worsening sensory impairment accompanied by radiographic evidence of neural compression or foreign material within the canal. In the present case, the decision to proceed with surgical decompression was based on failure of conservative measures and CBCT confirmation of canal obstruction.

The role of CBCT in endodontic diagnosis and complication assessment is well established. In this context, CBCT provided essential three-dimensional information regarding the spatial relationship between the extruded sealer and the inferior alveolar canal, allowing differentiation between periapical extrusion and true intracanal involvement. Such distinction is critical for endodontic decision-making, as minor extrusion adjacent to the canal may be managed non-surgically, whereas confirmed intracanal occupation carries a higher risk of persistent neural compromise [3].

Although the surgical phase of management lies outside routine endodontic practice, advances in digital planning and guided access have relevance to interdisciplinary care. In this case, guided piezoelectric access was used to limit bone removal and reduce the risk of additional mechanical insult to the nerve. We utilized piezoelectric instrumentation specifically to reduce surgical morbidity. However, surgical intervention remains a secondary option, reserved for cases where conservative management fails to resolve nerve compression [5, 6].

Following decompression, Leukocyte- and Platelet-Rich Fibrin (L-PRF) was utilized. While evidence for direct peripheral nerve regeneration using platelet concentrates is currently limited, the L-PRF served a dual purpose in this case:

Providing a biological “sleeve” for soft tissue coverage of the exposed nerve and acting as a scaffold for the “sticky bone” graft used to reconstruct the buccal cortical defect [9, 17].

Consequently, the observed neurosensory improvement cannot be attributed to a single intervention but rather to removal of the causative factor and subsequent resolution of neural compression.

Assessment of recovery relied on serial clinical neurosensory testing supplemented by pulp sensibility evaluation of adjacent teeth. While electric pulp testing does not directly measure nerve regeneration, changes in response thresholds may reflect restoration of inferior alveolar nerve conductivity and improved pulpal innervation, providing supportive but indirect evidence of functional recovery [8]. These findings must be interpreted cautiously and in conjunction with the overall clinical presentation. However, it is essential to note that in a pediatric patient, the potential for neural regeneration is naturally higher than in adults. The observed improvement is most likely attributed to the timely removal of the causative factor, thereby arresting the chemical and mechanical cascade of injury.

While the recovery of this 12-year-old patient is encouraging, it is difficult to isolate exactly which factor—the surgical decompression, the L-PRF, or the body’s natural healing over time—was most responsible for the neurosensory improvement. We must also be cautious about applying these results to adults or different materials, as pediatric anatomy and the unique properties of bio-ceramic sealers played a significant role here. This case should not be seen as a call for routine surgery after every sealer extrusion. Rather, it highlights a successful strategy for those difficult cases where conservative therapy fails and imaging clearly shows the nerve is obstructed. Larger clinical studies will be essential to help us refine exactly when to move from observation to surgical intervention.

This case demonstrates the use of digitally guided piezoelectric decompression for the removal of extruded bio-ceramic sealer from the inferior alveolar canal in the presence of persistent neurosensory disturbance. Controlled surgical access and removal were associated with gradual neurosensory improvement and radiographic resolution.

## Supplementary Information

Supplementary Material 1

## Data Availability

The datasets used and/or analysed during the current study are available from the corresponding author on reasonable request.

## Abbreviations

CBCT: Cone-beam computed tomography
CAD/CAM: Computer aided design/ computer aided manufacturing
L-PRF: Leukocyte- and platelet-rich fibrin
EPT: Electric pulp testing
